# Effects of pituitary-specific overexpression of FSHα/β on reproductive traits in transgenic boars

**DOI:** 10.1186/s40104-017-0208-y

**Published:** 2017-10-25

**Authors:** Wenting Li, Yujun Quan, Mengmeng Zhang, Kejun Wang, Muzhen Zhu, Ye Chen, Qiuyan Li, Keliang Wu

**Affiliations:** 1 College of Animal Science and Technology, China Agricultural University, Beijing, 100193 China; 2 College of Animal Science and Veterinary Medicine, Henan Agricultural University, Zhengzhou, 450002 China; 3 Institute of Zoology, Chinese Academy of Sciences, Beijing, 100101 China; 4 The Department of Animal Husbandry, Rongchang Campus, Southwest University, Rongchang, Chongqing, 402460 China; 5 State Key Laboratory for Agrobiotechnology, China Agricultural University, Beijing, 100193 China

**Keywords:** Boar, FSHα/β, Reproductive traits, Transgene

## Abstract

**Background:**

Follicle-stimulating hormone (FSH) is a gonadotropin synthesized and secreted by the pituitary gland. FSH stimulates follicle development and maturation in females. It also plays an important role in spermatogenesis in males, including humans and mice. However, the effects of FSH on male pigs are largely unknown. In this study, we generated transgenic pigs to investigate the effects of FSHα/β overexpression on reproductive traits in boars.

**Results:**

After five transgenic F_0_ founders were crossed with wide-type pigs, 193 F_1_ animals were obtained. Of these, 96 were confirmed as transgenic. *FSHα* and *FSHβ* mRNAs were detected only in pituitary tissue. Transgenic boars exhibited significantly higher levels of *FSHα* and *FSHβ* mRNA, serum FSH, and serum testosterone, compared to full-sib non-transgenic boars. Significant increases in testis weight, vas deferens diameter, seminiferous tubule diameter, and the number of Leydig cells were observed, suggesting that the exogenous FSHα/β affects reproductive traits. Finally, transgenic and non-transgenic boars had similar growth performance and biochemical profiles.

**Conclusions:**

Pituitary-specific overexpression of *FSHα/β* genes is likely to impact reproductive traits positively, as indicated by enhancements in serum testosterone level, testis weight, the development of vas deferens, seminiferous tubules, and Leydig cells in transgenic boars. A high level of serum FSH induces secretion of serum testosterone, possibly by boosting the number of Leydig cells, which presumably increases the libido and the frequency of sexual activity in transgenic boars. Our study provides a preliminary foundation for the genetic improvement of reproductive traits in male pigs.

## Background

Follicle-stimulating hormone (FSH) is a gonadotropin and glycoprotein polypeptide hormone with a mass of 35.5 kDa [1]. As a member of the glycoprotein hormone superfamily, it consists of two subunits (α and β) that combine non-covalently to form an active heterodimer, as is also the case for luteinizing hormone (LH), thyroid-stimulating hormone (TSH), and human chorionic gonadotropin (hCG) [1]. The synthesis and secretion of FSHα and FSHβ is regulated by gonadotropin-releasing hormone (GnRH). FSHβ is also regulated by inhibin, leptin, and activins derived from brain, pituitary, placenta, and other tissues [2–4]. In females, FSH plays a key role in antral follicle development and stimulates preovulatory follicular growth in cooperation with LH [5, 6]. In males, FSH is required for the mitotic division of germ cells, and together with testosterone, is involved in spermatocyte maturation and spermatogenesis [7].

Transgenic mouse models incorporating human *FSHα* and *FSHβ* genes have been used to study the effect of FSH on reproductive function [8]. In transgenic mice carrying a 10 kb human *FSHβ* construct, the inserted gene is highly and specifically expressed in pituitary tissue and the mice exhibit normal fertility [9, 10]. *FSH*-null (knockout) male mice are fertile and sire normal-sized litters, although they show reductions in epididymal sperm number, sperm motility, and testicle size, while female knockouts are infertile [5]. *FSHβ* has been verified to be an important gene controlling litter size in Chinese Erhualian pigs, one of the most prolific pig breeds in the world [11]. In transgenic mice exhibiting pituitary-specific overexpression of the Chinese Erhualian *FSH* gene, ovulation rate and litter size increase markedly [12].

F_0_ transgenic pigs, in which *FSHα/β* expression is pituitary-specific, were generated previously [13]. In this study, we obtained 193 F_1_ transgenic animals derived from five F_0_ founders crossed to wild-type Large White pigs. Integration of the exogenous *FSHα/β* genes and their expression were confirmed. Since genetic improvements are more efficiently transferred by males than by females in pig breeding, we focused on the effects of FSHα/β on reproductive traits in boars. As is typical in reproductive trait studies, multiple traits were assessed, including semen volume, sperm quality parameters, sperm per ejaculate, epididymis weight, reproductive tract weight, and seminiferous tubule diameters (Animal QTL database) [14]. Hormone assays and histological analyses were performed to investigate the effects of exogenous FSH expression on the reproductive traits of male offspring. In addition, the health status of transgenic pigs was evaluated based on growth and various biochemical criteria. The results are directly relevant to strategies for improving the fecundity of multiparous mammals.

## Methods

### Generation of transgenic pigs

**Fig. 1:**
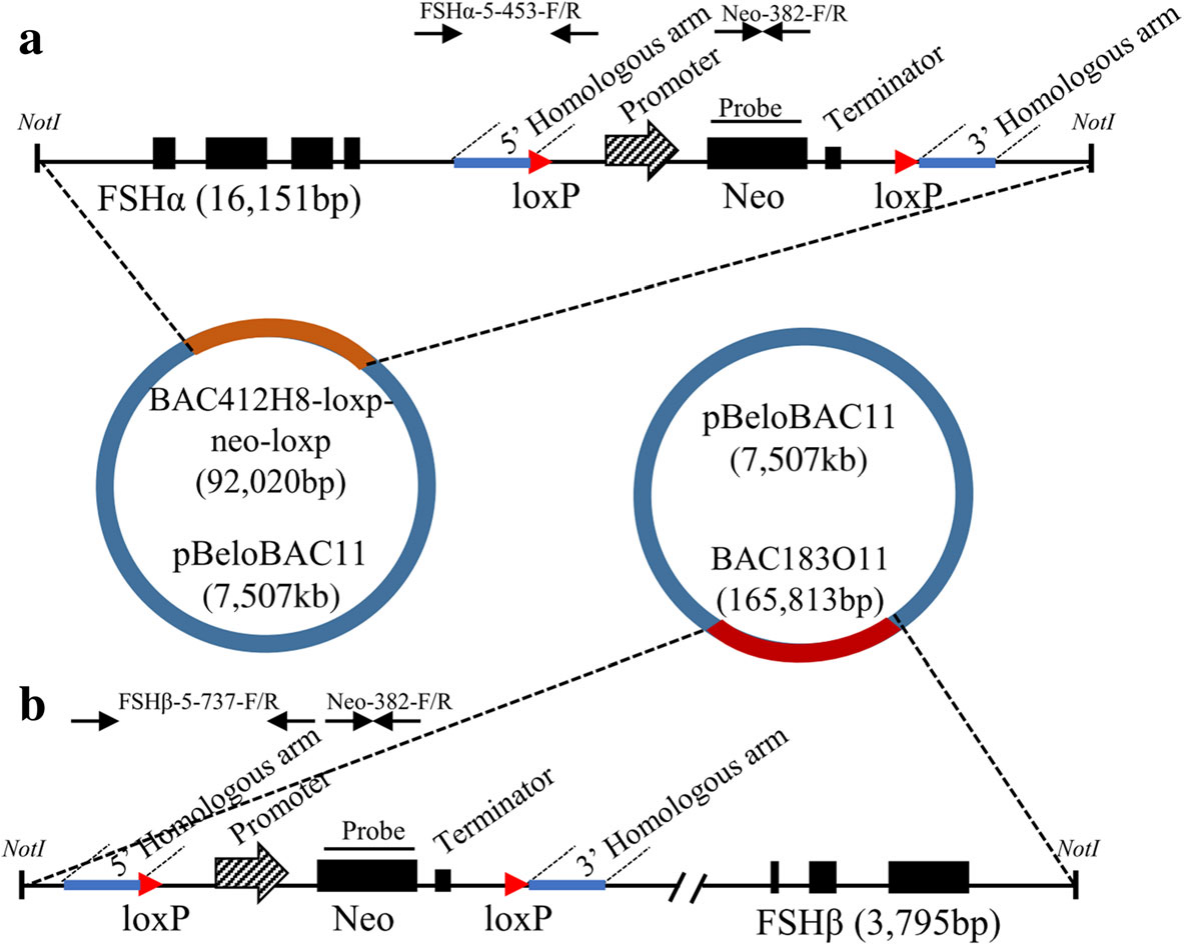
Schematic view of *FSHα* and *FSHβ* expression vectors. The vectors include the complete DNA sequences of the *FSHα* and *FSHβ* genes, along with the *Neo* gene and its promoter and terminator. Solid boxes represent exons. The red arrows represent *LoxP*, and the homologous arms are represented in blue. PCR primers (*FSHα*-5-453-F/R, *FSHβ*-5-737-F/R and *Neo*-382-F/R) are represented by black arrows. Southern blot probes are indicated by the label “probe”. *NotI* was the restriction enzyme cutting site

BAC DNA used for the production of transgenic animals in this study was described previously [12]. BAC clones for *FSHα* (BAC412H8) and *FSHβ* (BAC183O11) were isolated from a BAC library constructed using genomic DNA from a male Erhualian pig [15]. The LoxP-neo-LoxP cassette was introduced into two BAC constructs (*FSHα* and *FSHβ*) by homologous recombination (Fig. 1). BAC DNAs were linearized with *NotI* and co-transfected into fetal fibroblast cells. Positive cells were used as donors to produce transgenic founder pigs following standard procedures [16]. Transgenic F_0_ pigs were mated with non-transgenic Large White pigs to produce F_1_ pigs.

### Identification of transgenic pigs and detection of gene expression

Transgenic pigs were identified by PCR and Southern blot using genomic DNA extracted from ear tissue. Three pairs of primers, *FSHα*-5-453-F/R (453 bp product), *FSHβ*-5-737-F/R (737 bp product) [13], and *Neo*-382-F/R (382 bp product), were used to amplify *FSHα, FSHβ*, and *Neo*, respectively. PCR products were digested with *AvaII* and *PstI* prior to gel electrophoresis. The primers *Neo*-382-F/R (forward 5′-GTTGTCACTGAAGCGGGAAG-3′ and reverse 5′-CACAGTCGATGAATCCAGAAAA-3′) were used to generate a digoxigenin (DIG)-labeled probe for the Southern blot assay (Roche Diagnostics, Mannheim, Germany). All primers were synthesized by the Sangon Company (Shanghai, China).

Three F_1_ transgenic (Tg) boars and three non-transgenic (NTg) full-sib boars were slaughtered at approximately 300 d of age. Tissue samples from hypothalamus, pituitary, testis, epididymis, vas deferens, seminal vesicle, prostate, Cowper’s gland, heart, liver, spleen, lung, kidney, and pancreas were collected, rapidly frozen in liquid nitrogen, and stored at −80 °C. Tissue-specific expression of the *FSHα* and *FSHβ* transgenes was determined by reverse transcription PCR (RT-PCR) and quantified by real-time PCR [17]. Total RNA was extracted using an animal total RNA extraction kit according to the manufacturer’s instructions (Tiangen, Beijing, China). cDNA synthesis was performed with 1 μg total RNA following the protocol accompanying the FastQuant RT Kit (Tiangen, Beijing, China). *GADPH* expression was used for normalization. The specific primers used for quantifying expression were: *FSH-α* (forward: 5′-GGGTGCCCCAATCTATCAGTG-3′, reverse: 5′-GTGGCATTCGGTGTGGTTCTC-3′), *FSH-β* (forward: 5′-CACCCCAAGATGAAGTCGCTG-3′, reverse: 5′-GCCAGGTACTTTCACGGTCTCG-3′), and *GADPH* (forward: 5′-GTTTGTGATGGGCGTGAAC-3′, reverse: 5′-ATGGACCTGGGTCATGAGT-3′).

### Phenotype measurements

#### Body weight

Body weight of 20 F_1_ pigs (10 Tg and 10 NTg half-sib individuals) was recorded at the ages of 1 d (birth weight), 10 d and 21 d (weaning weight), 60 d, 90 d, and 150 d.

#### Serum biochemistry

Serum was separated from blood samples obtained from F_1_ pigs (5 Tg and 5 of NTg half-sib individuals) at 300 d, 307 d, and 315 d. The following compounds were measured: glucose (GLU), urea (UREA), creatinine (CREA), blood urea nitrogen/creatinine (BUN/CREA), phosphorus (PHOS), calcium (CA), total protein (TP), albumin (ALB), globulin (GLB), alanine aminotransferase (ALT), alkaline phosphatase (ALKP), γ-glutamyl transpeptidase (GGT), cholesterol (CHOL), triglyceride (TRIG), amylase (AMYL), lipase (LIPA), and creatine kinase (CK). All assays were conducted at Beijing Tianzewanwu Veterinary Hospital, China.

#### Hormone assays

Serum from three pairs of randomly chosen Tg and NTg full-sib boars was collected 3 times within one week at ~300 d of age. Levels of FSH, LH, testosterone, and estradiol (E2) were measured in triplicate using a standard radioimmunoassay. Assays were conducted at the Beijing North Institute of Biological Technology, China.

#### Assessment of sperm quality

Semen collection and quality assessments were performed as described [18]. Briefly, semen was collected from five pairs of Tg and NTg half-sib boars at an approximate age of 300 d. Three successive collections were performed at 7-day intervals. Semen volume was measured using graduated semen collection jars. Sperm concentration and motility were analyzed using the Sperm Quality Analyzer (Beijing, China). Total sperm number per ejaculate was calculated using the formula: sperm concentration × semen volume. The fraction of sperm exhibiting teratospermia, intact acrosomes, and normal mitochondrial function was assessed using methods described previously [19]. Seminal plasma quality was assessed by measuring levels of zinc, fructose, neutral α-glucosidase (NAG), and acid phosphatase (ACP), using a ChemWell BRED Analyzer (Guangdong, China) at the Beijing North Institute of Biological Technology.

#### Histological analysis

After slaughter, testes and epididymis were isolated and weighed. Testes tissue and vas deferens was fixed in 4% paraformaldehyde, embedded in paraffin, and sectioned. Tissue sections were stained with hematoxylin-eosin (H&E) and observed with a light microscope (Nikon, Japan). The diameters of vas deferens and seminiferous tubules were measured in ~30 fields. Leydig cells were counted in ~10 fields for each pig at 200× magnification and the average value was calculated.

### Statistical analysis

Student’s *t*-test was performed using SPSS Statistics (IBM Corporation, USA). All values are presented as mean ± standard error (SEM). *P* < 0.05 was the threshold for statistical significance.

## Results

Transgenic pigs exhibiting pituitary-specific overexpression of the *FSHα/β* genes were generated using the BAC DNAs (*FSHα* and *FSHβ*) shown in Fig. 1. Five F_0_ transgenic animals (two boars and three sows), in which both BACs were intact, were identified by PCR and Southern blot analysis, as described by Bi [13].

### Integration and expression of exogenous *FSH*

**Fig. 2:**
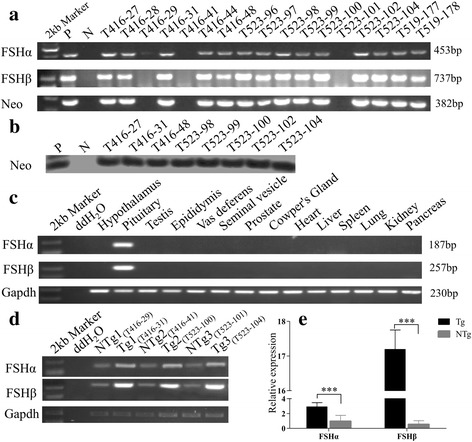
Identification of exogenous *FSHα/β* insertion and expression analysis. (**a**) Identification of F_1_ transgenic pigs by PCR using DNA obtained from ear tissue. P, a single F_0_ transgenic pig as positive control; N, a single non-transgenic Large White pig as negative control; T416–27, 28, 29, 31, 41, T523–96, 97, 98, 99, 100, 101, 102, 104, and T519–177, 178 are identifiers for F_1_ transgenic pigs. (**b**) Southern blot for transgenic pig identification. The *Neo* gene in transgenic pigs was detected using the probe shown in Fig. 1. DNAs were digested with *AvaII* and *PstI* to generate a target fragment of 463 bp. (**c**) RT-PCR analysis of *FSHα* and *FSHβ* from pituitary and 13 other tissues. *GADPH* was used as a control. (**d**) RT-PCR analysis of *FSHα* and *FSHβ* expression using mRNA from the pituitaries of six transgenic pigs. (**e**). *FSHα* and *FSHβ* mRNA expression levels in the pituitaries of Tg and NTg pigs analyzed using qPCR. Relative expression was calculated relative to *β-actin* (reference gene). Values are expressed as means ± SEM. ***, *P* < 0.001

Five founders were crossed with wild-type Large White pigs to obtain 193 F_1_ progenies, of which nearly half (43 boars and 53 sows) were positive for the exogenous *FSHα, FSHβ* and *Neo* genes, as determined by PCR (Fig. 2a). The *Neo* gene was also detected by Southern blot in all 96 F_1_ pigs (Fig. 2b). These data confirm that the integrated *FSHα, FSHβ* and *Neo* genes were transmitted to both male and female F_1_ pigs with the expected Mendelian ratio.

To determine whether the exogenous *FSHα* and *FSHβ* genes in the F_1_ transgenic pigs were expressed in a tissue specific manner, *FSH* mRNA from pituitary gland and 13 other tissues was subjected to RT-PCR. *FSHα* and *FSHβ* expression was observed only in pituitary tissue (Fig. 2c). Because this experiment does not distinguish between contributions made by exogenous and endogenous *FSH* genes, *FSHα* and *FSHβ* expression in the pituitary glands of three pairs of full-sib transgenic and non-transgenic boars was compared by RT-PCR (Fig. 2d), and total *FSH* mRNA expression was quantified in the same samples using qPCR (Fig. 2e). As expected, mRNA levels of both *FSHα* and *FSHβ* were significantly higher in transgenic animals (*P* < 0.001).

### Serum concentrations of FSH, LH, testosterone, and E2

**Fig. 3:**
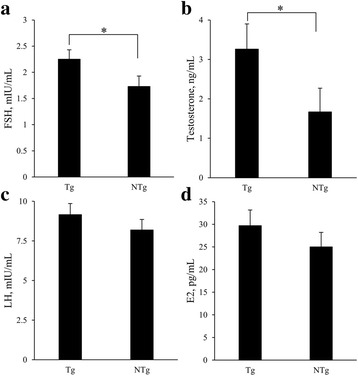
Hormone assays. (**a**) Serum FSH, (**b**) testosterone, (**c**) LH, and (**d**) E2 levels in F_1_ pigs. All assays were conducted in triplicate. Bars represent means ± SEM. *, *P* < 0.05

To examine the effects of FSHα/β overexpression on hormone levels, FSH, LH, testosterone and E2 levels were compared in full-sib transgenic and non-transgenic boars at an approximate age of 300 d (Fig. 3). Serum levels of FSH were significantly higher in transgenic animals (2.25 ± 0.18 mIU/mL vs. 1.75 ± 0.20 mIU/mL, *P* < 0.05, Fig. 3a). Similarly, testosterone levels in transgenic boars were significantly higher than in non-transgenic boars (3.26 ± 0.64 ng/mL vs. 1.67 ± 0.60 ng/mL, *P* < 0.05, Fig. 3b). Although serum levels of both LH and E2 were higher in transgenic boars, the differences were not significant (LH: 9.16 ± 0.70 mIU/mL vs. 8.19 ± 0.67 mIU/mL, *P* > 0.05; E2: 29.71 ± 3.46 pg/mL vs. 25.00 ± 3.22 pg/mL, *P* > 0.05; Fig. 3c-d).

### Effect of FSH overexpression on reproductive traits

Several semen quality indicators and seminal plasma components were compared between transgenic and non-transgenic boars at ~300 d of age. No significant differences were observed in any of the seven semen quality indicators (*P* > 0.05, Table 1). Transgenic and non-transgenic boars exhibited similar values for all four seminal plasma components (*P* > 0.05, Table 2).

**Table 1:** Semen characteristics in transgenic and non-transgenic boars

Items	Tg	NTg	*P*-value
Semen volume per ejaculate, mL	218.75 ± 28.73	237.00 ± 29.54	0.079
Sperm concentration, 10^8^/mL	3.58 ± 0.09	3.46 ± 0.08	0.619
Total sperm per ejaculate, 10^8^	794.74 ± 28.35	832.78 ± 25.36	0.314
Sperm mobility, %	77.11 ± 2.63	73.57 ± 2.36	0.411
Teratospermia, %	8.23 ± 0.30	7.30 ± 0.38	0.764
Acrosome intactness, %	81.68 ± 0.25	81.34 ± 0.23	0.890
Normal mitochondria function, %	80.18 ± 1.52	82.22 ± 1.36	0.930

**Table 2:** Biochemical indicators for seminal plasma in transgenic and non-transgenic boars

Items	Tg	NTg	*P*-value
Seminal plasma zinc, μmol	0.76 ± 0.16	0.54 ± 0.15	0.248
Seminal plasma fructose, mIU	1.01 ± 0.05	0.95 ± 0.05	0.919
Neutral α-glucosidase, I﻿U	0.95 ± 0.06	0.70 ± 0.05	0.324
Acid phosphatase, μmol	236.90 ± 14.17	217.89 ± 12.67	0.406

**Fig. 4:**
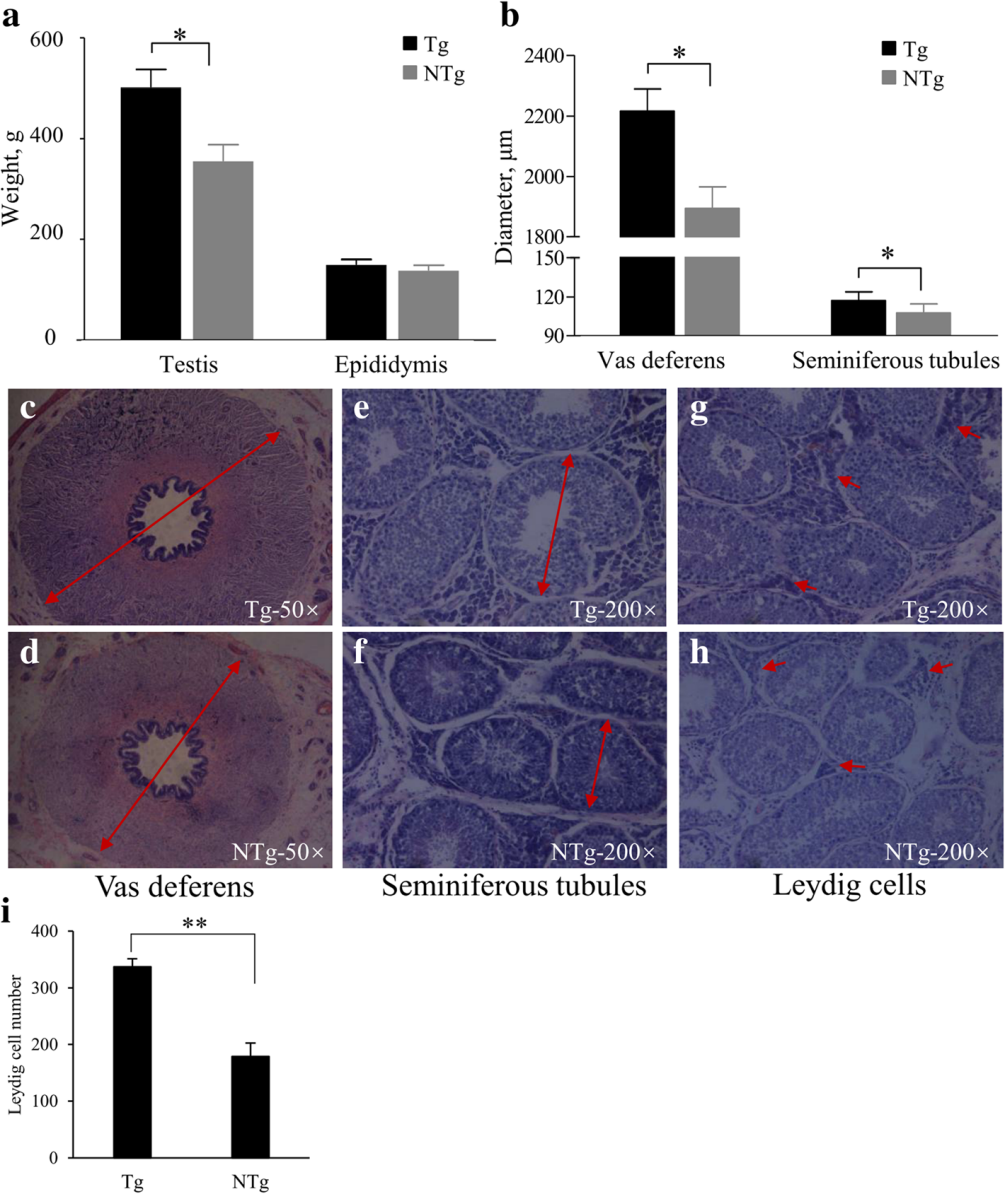
Histological assessment of reproductive tissue from F_1_ boars. (**a**) Comparison of testis and epididymis weight in Tg and NTg boars. (**b**) Vas deferens and seminiferous tubule diameters in Tg and NTg boars. (**c-i**) Histological sections of testis tissue. (**c-d**) Vas deferens at 50× magnification. Red arrows span the vas deferens diameter. (**e-f**) Seminiferous tubules at 200× magnification. Red arrows span tubule diameter. (**g-h**) Leydig cells at 200× magnification. Red arrows indicate Leydig cells between the seminiferous tubules. (**i**) The number of Leydig cells in Tg and NTg boars﻿. Data are expressed as means ± SEM. *, *P *< 0.05. **, *P* < 0.01

We also compared testis and epididymis characteristics between transgenic and non-transgenic boars. As shown in Fig. 4a, the testis weight in transgenic boars was significantly higher (501.6 ± 35.6 g vs. 355.2 ± 32.8 g, *P* < 0.05). Transgenic boars exhibited higher epididymis weight but the levels were statistically indistinguishable (149.6 ± 10.6 g vs. 138.0 ± 11.0 g, *P* > 0.05, Fig. 4a). Vas deferens and seminiferous tubule diameters were also compared. Interestingly, both diameters were significantly higher in transgenic boars (vas deferens, 2216.25 ± 173.24 μm vs. 1894.72 ± 270.86 μm, *P* < 0.001; seminiferous tubules, 117.30 ± 6.65 μm vs. 107.79 ± 6.79 μm, *P* < 0.001, Fig. 4b-f). Enlargement of the vas deferens occurred mainly in the muscular layer of the wall. Finally, the number of Leydig cells in transgenic boars was significantly higher than in non-transgenic boars (337.6 ± 14.3 vs. 178.9 ± 23.4, *P* < 0.01, Fig. 4g-i).

### Growth and biochemical analysis

**Fig. 5:**
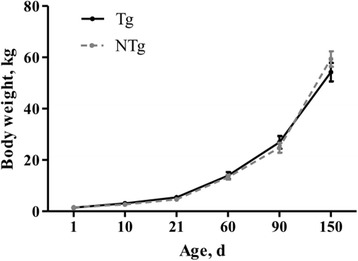
Growth of F_1_ Tg and NTg boars from birth to 150 d. Data is expressed as means ± SEM

Body weight at six growth stages (from birth to 150 d) was compared between transgenic and non-transgenic boars. There were no significant differences, although transgenic boar body weight was slightly higher from birth to 90 d, while non-transgenic boars exhibited higher body weight at 150 d (Fig. 5). In addition, no significant differences in blood chemistry were observed (Table 3). We conclude that the transgenic boars in this study exhibited no detectable health defects relative to wild-type controls.

**Table 3:** Blood biochemistry in transgenic and non-transgenic boars

Items	Tg	NTg	*P*-value
Glucose, mmol/L	4.58 ± 0.18	4.33 ± 0.16	0.334
Urea, mmol/L	6.87 ± 0.24	7.19 ± 0.21	0.785
Creatinine, μmol/L	118.42 ± 3.17	122.67 ± 2.83	0.0629
Blood urea nitrogen/creatinine	16.50 ± 0.52	15.80 ± 0.47	0.254
Phosphorus, mmol/L	2.28 ± 0.14	2.10 ± 0.12	0.0516
Calcium, mmol/L	2.29 ± 0.03	2.33 ± 0.03	0.580
Total protein, g/L	70.75 ± 0.36	70.27 ± 0.32	0.491
Albumin, g/L	32.50 ± 0.17	32.27 ± 0.16	0.526
Globulin, g/L	38.25 ± 0.19	38.00 ± 0.17	0.831
Albumin/ Globulin	0.84 ± 0.02	0.87 ± 0.02	0.545
Alanine aminotransferase, ﻿IU/L	59.17 ± 0.62	60.00 ± 0.56	0.809
Alkaline phosphatase, ﻿I﻿U/L	76.75 ± 4.61	82.93 ± 4.12	0.366
γ-glutamyl transpeptidase, I﻿U/L	32.92 ± 1.25	34.60 ± 1.22	0.802
Cholesterol, mmol/L	1.89 ± 0.07	1.80 ± 0.06	0.182
Triglyceride, mmol/L	0.59 ± 0.06	0.50 ± 0.06	0.406
Amylase, ﻿IU/L	434.03 ± 30.35	474.80 ± 27.14	0.617
Lipase, ﻿IU/L	23.50 ± 1.78	21.08 ± 1.63	0.496
Creatine kinase, IU/L	606.64 ± 18.65	591.08 ± 15.93	0.729

## Discussion

Pig fecundity is one of the most economically important traits in pig production. Because pig reproductive traits have low heritability [20], only a few candidate genes affecting pig reproduction have been identified, such as estrogen receptor 1 (*ESR1*) and *FSHβ* [11, 21]. Transgenic mice in which porcine FSH is overexpressed exhibit significantly increased female fertility [12]. In this study, we investigated the effects of porcine FSH on reproductive traits in male transgenic pigs.

In 193 F_1_ progenies, 96 transgenic pigs were identified. The transmission rate was 49.74%, consistent with ordinary Mendelian inheritance. FSH expression occurred in a pituitary-specific pattern (Fig. 2c), similar to results reported for FSHβ-overexpressing mice [12]. Because the exogenous and endogenous porcine FSHα/β are nearly identical in sequence, we could not distinguish between them using molecular methods. However, when total *FSHα/β* mRNA and serum FSH were compared in transgenic and non-transgenic pigs, transgenic animals exhibited significantly higher levels. These results suggest that pituitary-specific overexpression of FSH was successfully established in our transgenic pig model. While *FSHβ* mRNA increased approximately 10-fold in the transgenic animals, and *FSHα* mRNA increased about 3-fold, we observed only a modest increase in serum FSH levels (Figs. 2e and 3a). This is expected because serum FSH is a heterodimer, consisting of two subunits of FSHα and FSHβ, and FSH levels are probably limited by the lower level of *FSHα* mRNA expression (Fig. 2d-e) [1].

Male fertility is important in reproductive performance [22], and growing evidence suggests that FSH may be an important factor. In our study, the diameter of vas deferens and seminiferous tubules (Fig. 4b-f) increased with the increasing levels of serum FSH in transgenic boars (Fig. 3a). The enlargement of vas deferens mainly occurred in the muscular layer of the wall. In humans, the vas deferens wall is thinner after vasectomy [23]. We suggest that the thickened muscular layer of the vas deferens might affect sperm transportation and the ejaculation process, but the hypothesis has not yet been tested. Seminiferous tubule diameter correlates positively with semen quality parameters (sperm concentration, sperm motility, and total sperm per ejaculate) in rabbits [24]. In contrast, no improvement in semen quality was identified in transgenic boars in this study. In addition, semen quality in pigs does not change after treatment with FSH, although serum testosterone level increases [25]. Testosterone levels are enhanced in male mice that overexpress FSH [10]. In contrast, *FSH* and FSH receptor knockout mice have smaller testes and exhibit reduced numbers of germ and Leydig cells [5, 26]. In this study, we also observed that the serum testosterone level (Fig. 3b), testis weight (Fig. 4a) and the number of Leydig cells (Fig. 4g-i) increased in transgenic boars. The main function of Leydig cells is testosterone synthesis and secretion [27], and serum testosterone concentration is strongly related to libido in humans [28], rams [29], rats [30], and mice [31]. Testosterone also enhances libido, frequency of sexual acts, and sleep-related erections in humans [32]. If the underlying biology is similar in pigs, the increased number of Leydig cells in transgenic boars would be expected to increase testosterone levels and thereby enhance libido, increase the frequency of sexual activity, and increase the frequency of semen collection. Because our results indicate that overexpression of FSH increases serum testosterone levels in boars, the effect is likely to be an improvement in the downstream reproductive traits.

Finally, we evaluated whether the exogenous *FSHβ* gene exerts deleterious effects on the transgenic pigs. Body weight, levels of various biochemical components in blood plasma and semen plasma, and semen quality, were similar in transgenic and non-transgenic animals. This suggests that FSH overexpression has no detectable adverse impact on pig health.

## Conclusions

In summary, we successfully produced transgenic pigs in which exogenous *FSHα/β* genes were integrated and expressed at high levels in a pituitary-specific manner. The high level of serum FSH increases the level of serum testosterone, possibly by increasing the number Leydig cells. Higher levels of testosterone would be expected to enhance the libido and the frequency of sexual activity in transgenic boars. Nevertheless, augmented FSH levels did not improve semen quality, even though testis weight and seminiferous tubules diameter increased. Finally, the expression of exogenous *FSHα/β* genes resulted in no detectable adverse effects on growth or the overall health of transgenic boars.

## Abbreviations

ACP: Acid phosphatase
ALB: Albumin
ALKP: Alkaline phosphatase
ALT: Alanine aminotransferase
AMYL: Amylase
BUN/CREA: Blood urea nitrogen/creatinine
CA: Calcium
CHOL: Cholesterol
CK: Creatine kinas
CREA: Creatinine
DIG: Digoxigenin
E2: Estradiol
ESR1: Estrogen receptor 1
FSH: Follicle-stimulating hormone
FSHα: Follicle-stimulating hormone subunit α
FSHβ: Follicle-stimulating hormone subunit β
GGT: γ-glutamyl transpeptidase
GLB: Globulin
GLU: Glucose
GnRH: Gonadotropin-releasing hormone
hCG: Human chorionic gonadotropin
LH: Luteinizing hormone
LIPA: Lipase
NAG: Neutral α-glucosidase
PHOS: Phosphorus
TP: Total protein
TRIG: Triglyceride
TSH: Thyroid-stimulating hormone
